# Midsagittal Anatomy of Lumbar Lordosis in Adult Egyptians: MRI Study

**DOI:** 10.1155/2014/370852

**Published:** 2014-08-18

**Authors:** Abdelmonem A. Hegazy, Raafat A. Hegazy

**Affiliations:** ^1^Anatomy Department, Faculty of Medicine, Zagazig University, Zagazig 44519, Egypt; ^2^Pathology Department, Faculty of Medicine, Zagazig University, Zagazig 44519, Egypt

## Abstract

Despite the increasing recognition of the functional and clinical importance of lumbar lordosis, little is known about its description, particularly in Egypt. At the same time, magnetic resonance imaging (MRI) has been introduced as a noninvasive diagnostic technique. The aim of this study was to investigate the anatomy of the lumbar lordosis using midsagittal MRIs. Normal lumbar spine MRIs obtained from 93 individuals (46 males, 47 females; 25–57 years old) were evaluated retrospectively. The lumbar spine curvature and its segments “vertebrae and discs” were described and measured. The lumbar lordosis angle (LLA) was larger in females than in males. Its mean values increased by age. The lumbar height (LH) was longer in males than in females. At the same time, the lumbar breadth (LB) was higher in females than in males. Lumbar index (LI = LB/LH × 100) showed significant gender differences (*P* < 0.0001). Lordosis was formed by wedging of intervertebral discs and bodies of lower lumbar vertebrae. In conclusion, MRI might clearly reveal the anatomy of the lumbar lordosis. Use of LI in association with LLA could be useful in evaluation of lumbar lordosis.

## 1. Introduction

There is an increasing recognition of the functional and clinical importance for lumbar lordosis [1]. It is the key postural component in maintaining sagittal balance [2]. Affection of lumbar lordotic curve often results in sagittal spinal imbalance causing low back pain that represents one of the leading causes of disability [3]. Therefore, there is a need for accurate reconstruction of the lordotic curvature [2]. However, the current knowledge base for such reconstruction and spinal surgery is insufficient [4]. The normal range of lumbar lordosis is so wide (30 to 80°) that it becomes difficult to determine its value for an individual [2]. Unfortunately, the available data measuring the lumbar spine curvature using MRI are still limited, particularly in Egypt. Such data are used in assessing postural abnormalities [2]. Also, determining the size of the intervertebral disc and lumbar body vertebra is needed for the interbody fusion and artificial disc replacement [5]. Studies on the cadaver are subject to distortion because of postmortem tissue changes [6]. Meanwhile, the development of MRI has greatly enhanced understanding of the living human anatomy [7].

Aim of the study was to illustrate the normal midsagittal lumbar lordosis in adult Egyptians, its morphology and values using magnetic resonance imaging (MRI), and to evaluate the role of lumbar spine segments “vertebrae and intervertebral discs” in its formation. The established database could be useful as reference values for the evaluation of lumbar bodies and discs in symptomatic patients.

## 2. Material and Methods

### 2.1. Subjects and MRI

A retrospective study was done for cases referred to the Diagnostic Radiology Department, Zagazig University Hospitals, in the period between January 2011 and June 2014. The data about the age and sex were recorded. MRI of the lumbosacral region for each case was studied. It was performed for the subject in the routine supine position with the hips and knees flexed. The images were obtained for various reasons such as soft tissue injuries, muscle pain, and low back pain. The selected cases were 93 in number, showing normal findings on T1 and T2 images without any change in the intervertebral discs and the surrounding bones according to the reading of the radiologist. The images were excluded if a fracture, congenital anomaly (such as lumbarisation and sacralisation), previous lumbar surgery, or pathology affecting the anatomy of the vertebrae and intervertebral discs was present. Also, the preliminary coronal scans were examined to ensure that the spine did not show significant scoliosis or any other rotation.

### 2.2. Protocol of MRI

The lumbar spine was examined with the use of a 1.5 Tesla scanner. T1-weighted images in the sagittal plane were obtained using a single spin-echo technique with a repetition time (TR) of 400 milliseconds and echo time (TE) of 8 milliseconds. Repetition time (TR) for T2-weighted images was 2800 milliseconds while for echo time (TE) it was 120 milliseconds. Slice thickness was 4 mm. The field of view (FOV) used was 25–30 cm which readily contained the lumbar spine with the last thoracic vertebra and a part of the sacrum.

### 2.3. Measurements

**Figure 1:**
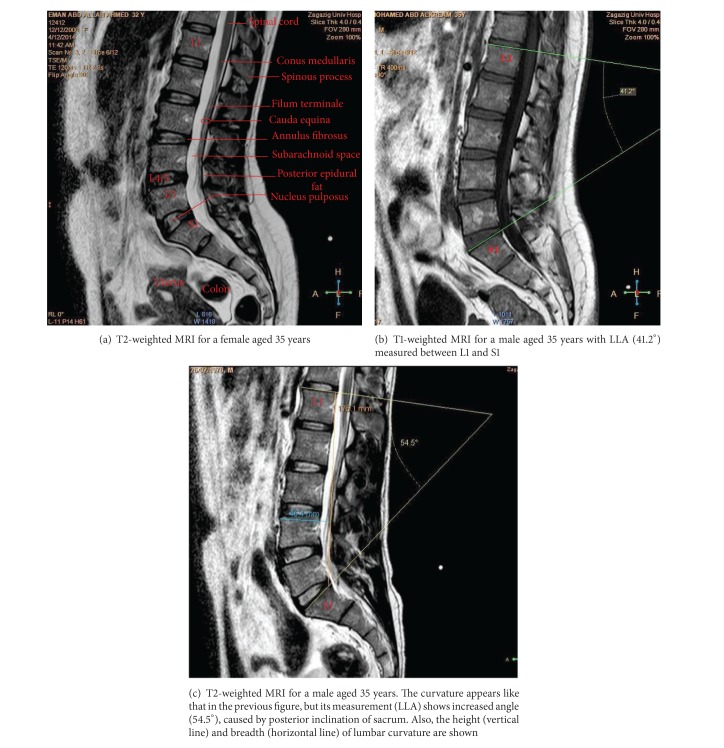
Sagittal MRIs showing a gender difference in curvature of lumbar spine.

**Figure 2:**
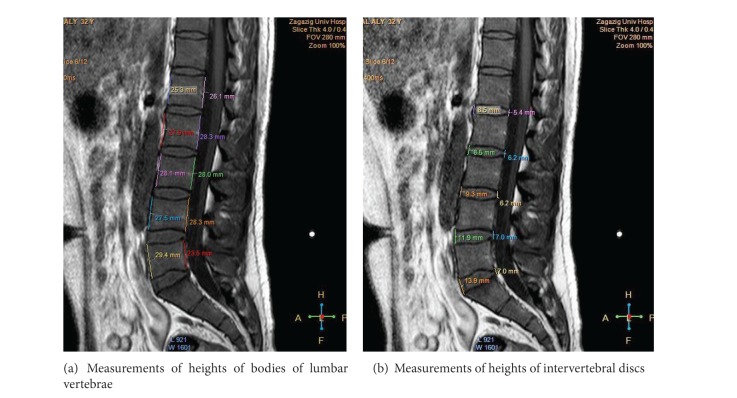
Sagittal T1-weighted MRI of a male aged 32 years showing measurements of lumbar spine segments.

All MRIs were examined in the midsagittal plane. Confirmation that the resulting images were truly midline for all lumbar segments was determined from the presence of the spinous processes and clear demarcation of the spinal cord (Figure 1(a)) [8]. Twenty-three anatomical parameters were measured for each case (Table 1). Each measurement was recorded twice by each author, one from sagittal T1-weighted MRI and the other from T2-weighted MRI. This procedure was performed on two different days. The average of the readings for each parameter was used in the final calculation of the statistics. The angle of lumbar curvature was measured according to the modified Cobb's method (Table 1, Figure 1(b)) [9]. Also, the height (LH) and breadth (LB) of the lumbar curvature were recorded (Figure 1(c)). Metric measurements included the anterior and posterior heights for each one of the five lumbar vertebrae (L1 to L5) and the intervertebral discs (L1/2 to L5/S1) (Figures 2(a) and 2(b)). All measurements were taken to the nearest 0.1 mm.

### 2.4. Statistical Analysis

First, the number of males and females was calculated. Then, each gender group was arranged into two age groups; the first group included ages from 25 to 41 years while the second one ranged from 42 to 57 years. This was followed by determining the mean age (±SD) of individuals for each group.

Second, we calculated the mean values (m) of lumbar lordosis angle (LLA), height (LH), and breadth (LB) for lumbar spine curvature and anterior and posterior heights of vertebrae (AL and PL) and intervertebral discs (AD and PD) for each group.

Third, the data were analyzed for reliability. The data were analyzed for inter- and intraobserver reliability using the interclass correlation coefficient (ICC). A reliability greater than or equal to an ICC of 0.75 (*P* < 0.05) was considered highly reliable [10].

Fourth, the following indices were determined.

- Lordosis index (LI) was calculated as the ratio of the breadth (LB) and height (LH) of the lumbar spine, as LI = LB/LH × 100 [11].
- Wedge index (WI) for each lumbar segment was calculated as the ratio of the anterior height to the posterior height [12] as follows.
  - Lumbar vertebral index = AL/PL × 100,
  - Intervertebral disc index = AD/PD × 100.

A vertebral body or disc with WI more than 100 was considered as a wedged (lordotic) segment. At the same time, the index less than 100 was a wedged segment in the opposite side (kyphosis); and that equaled 100 was a neutral “square” structure. Then, the mean values (m) of the indices for each group were calculated.

Finally, the obtained data were scrutinized, tabulated, and statistically analyzed, using maximum and minimum values, range (R), mean (m), difference between means of two groups (MD), standard deviation (SD), and 95% confidence intervals (CI) of mean. The existence of significant differences between the means for the gender and the age groups was analyzed by using independent Student's* t*-test. A *P* value <0.05 was considered to be statistically significant.

## 3. Results

### 3.1. Ages and Numbers

There were 46 males (M) and 47 females (F). Their ages ranged from 25 to 57 years. The first age group (G1) included 26 males and 20 females, while the second group (G2) included 20 males and 27 females (Table 2).

### 3.2. Morphological MRI Findings

**Figure 3:**
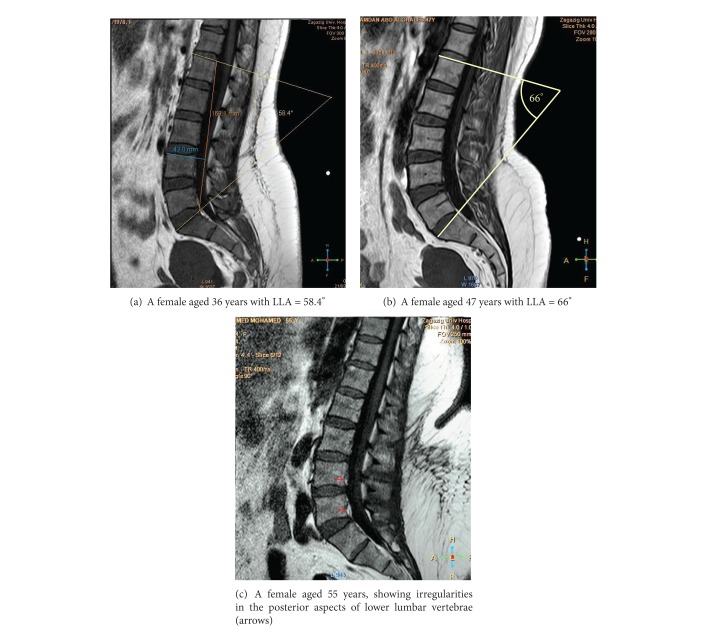
Sagittal T1-weighted female MRIs showing an increase in curvature of lumbar spine with aging.

The lumbar spine presented a posterior concavity “lordosis.” The lordosis was noticed to be more obvious in females than in males (Figures 1(a) and 1(b)) and increased by age (Figures 3(a), 3(b), and 3(c)). The lumbar spine comprised five vertebrae and five intervertebral discs. The vertebral bodies appeared on sagittal MRI as square masses separated by wedged elliptical intervertebral discs. The bodies demonstrated a low-signal outer rim surrounding the high-signal cancellous bone. The lumbar endplates were concave, while that of the upper surface of the sacrum was more or less flat. Meanwhile, the intervertebral discs had slightly less signal than the adjacent vertebral bodies; each disc was shown to consist of a central part, the nucleus pulposus, and a peripheral part, the annulus fibrosus, well differentiated on T2-weighted images. The discs increased in size in a craniocaudal direction. The maximum concavity of lumbar lordosis was noticed opposite to the upper edge of the fourth lumbar vertebra (Figures 1(c) and 3(a)). The height of fifth intervertebral disc (L5/S1) appeared to be markedly increased anteriorly, causing posterior inclination of the sacrum (Figures 2-3).

### 3.3. Inter- and Intraobserver Agreement

The values obtained at the different days by the same and each author were in close agreement with one another. The interclass correlation coefficient and the intraobserver agreement ranged from 0.90 to 0.97 and 0.95 to 0.98, respectively.

### 3.4. Measurement of Lumbar Lordosis Angle and Index

The values obtained for the angle of lumbar lordosis (LLA) ranged from 30° to 67°. Its mean in females (52.20°) was larger than in males (41.98°). This difference was considered to be extremely statistically significant (*P* value <0.0001). The angle increased by age, in both sexes. In males, its mean increased from 39.12° to 45.70° and in females from 50.03° to 53.81°, for the first and second age groups, respectively. Also, the lumbar height (LH) showed a significant increase in males (m: 168.08 mm) compared to that in females (m: 156.39 mm), with *P* value <0.0001. There was LH decrease in both sexes by age; means in males decreased from 170.39 mm in the first age group to 165.09 mm in the second group and in females from to 159.42 in the first group to 154.15 mm in the second group. At the same time, LB was slightly increased in females (m: 45.73 mm) compared to that in males (m: 44.02 mm), with *P* value =0.0553. On calculating the LI, there was a significant difference in its means between males (m: 26.26) and females (m: 29.34), with *P* value <0.0001 (Table 3).

### 3.5. Measurement of the Vertebral Body

**Figure 4:**
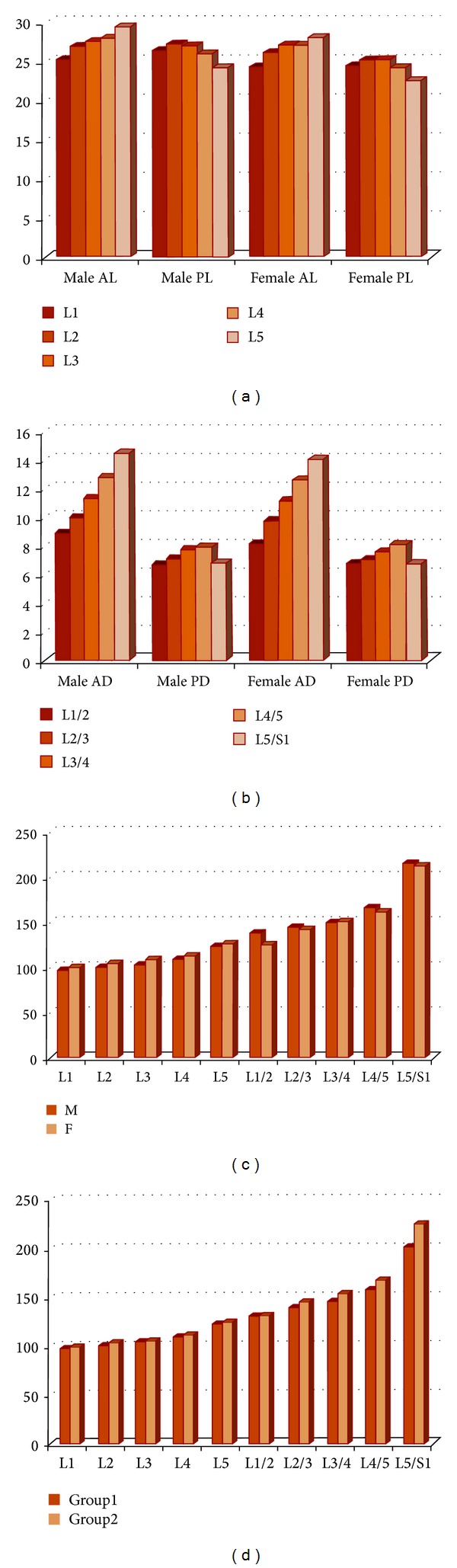
Graphs showing the differences in the mean values: (a) vertebral body heights (mm) in total investigated cases of males and females, (b) intervertebral disc heights (mm) in total investigated cases of males and females, (c) indices of wedging of lumbar spine segments in the investigated cases of males and females. Number 100 indicates the base line; above it is lordotic and below it is kyphotic segment; (d) indices of wedging of lumbar spine segments in the investigated groups of ages.

The anterior height (AH) of lumbar vertebral bodies in both sexes increased in a craniocaudal direction. Its mean for L1 vertebra was 25.23 mm and 24.18 mm in males and females, respectively. The value increased to reach 29.31 mm and 27.88 mm for L5 vertebra of males and females, respectively. In regard to the posterior height (PH), there was an increase in its mean in males from L1 (m: 26.30 mm) to L2 (m: 27.13 mm), followed by a slight and gradual decrease to reach L5 (m: 24.09 mm). The PH in females showed the same trend of the male PH, but the change in the values occurred at L3 instead of L2. All investigated dimensions of male vertebrae were greater than those of females, with variable *P* values (Figure 4(a); Table 4).

### 3.6. Measurement of the Intervertebral Disc

The lumbar disc heights generally increased toward the lower lumbar levels, except for the posterior height of L5/S1. The mean of anterior disc height (AD) was 8.91 mm and 8.11 mm for the first disc (L1/2) in males and females, respectively. Then, it increased gradually till it reached the last disc (L5/S1) where its value was 14.41 mm and 13.97 mm in males and females, respectively. On the other hand, the mean of posterior disc height (PD) of L1/2 was 6.60 mm in males and 6.69 mm in females; then, it increased gradually till the L4/5, where it reached its maximum values about 8.0 mm in both sexes. Then, the PD of L5/S1 decreased to reach about 7.0 mm in both sexes. Despite the increased disc dimensions in males compared to those in females in most cases, these differences were not statistically significant (Figure 4(b); Table 5).

### 3.7. Assessment of the Wedging of Lumbar Spine Segments

Investigation of lumbar indices (WI) in males showed that the lumbar bodies presented kyphotic wedging (WI < 100) at L1 and tended to be neutral “square” (WI = 100) at L2 and then were followed from L3 to L5 by a progressive lordotic bent (WI > 100), with variable *P* values between the two age groups. Female lumbar WI showed that lordotic trend began as high as L2 (Figure 4(c); Table 6).

The wedging of the intervertebral discs showed a lordotic trend (WI > 100) at all levels and an increase from the L1/2 (m: 137.02 for males and 124.68 for females) to the L5/S1 disc (m: 214.85 for males and 212.43 for females). The increase was in a gradient manner from L1/2 till L4/5 and then was followed by marked increase at L5/S1. The WI of discs showed no statistically significant difference between the two sexes. In regard to bodies of lumbar vertebrae, the WI means were higher in females than in males, with statistically significant differences, particularly in the second age group (Figure 4(c); Table 6). At all levels of lumbar segments, there was an increase in the mean values of WI by age, which appeared in the second age group in comparison with the first one. The difference was highly significant at the last disc “L5/S1” (*P* value =0.0024) (Figure 4(d); Table 7).

## 4. Discussion

Lumbar lordosis is the inward (ventral) curvature of the lumbar spine [13]. It is a key factor in maintaining sagittal balance or “neutral upright sagittal spinal alignment” which represents a postural goal for surgical, ergonomic, and physiotherapeutic intervention [2]. The normal range of LLA in the current study was 30° to 67°. The recorded range of LLA differed from that recorded in other studies, using radiographs in their assessment. Jackson and McManus [14] described values which ranged from 31° to 88°; and Damasceno et al. [15] reported a range from 33° to 89°. Our data showed an increased LLA in females (m: 52.20°) than in males (m: 41.98°), with *P* value <0.0001. Murrie et al. [16] agreed with the current results that lumbar lordosis is more prominent in females but they were unable to demonstrate any significant variation in lordosis with age. Stagnara et al. [17] argued that females apparently had greater lumbar lordosis owing to their greater buttock size. Another explanation for increased lordosis in females is the number of pregnancies. Nourbakhsh et al. [18] stated that the degree of lumbar lordosis was positively related with the number of pregnancies. During the later months of pregnancy, with the increase in size and weight of the fetus, women tend to increase the posterior lumbar concavity in an attempt to preserve their center of gravity [19]. Our results showed that LLA also increased by aging in both sexes, more markedly in males (m: 39.12° and 45.70° for the first and second age groups, resp.) than in females (m: 50.03° and 53.81° for the first and second age groups, resp.). These findings are in general in agreement with that of Tüzün et al. [20] who stated that lumbar lordosis and thoracic kyphosis are increased with age. Lee et al. [21] recognized a difference between younger and older subjects; but they accounted this difference to the disparity in flexibility or function of body parts. With lumbar hyperlordosis, the middle thoracic vertebrae tend to be more wedged, and the lumbar vertebrae tend to be more reverse-wedged [22]. Ghandhari et al. [23] agreed that lumbar lordosis and thoracic kyphosis are correlated, so that lumbar lordosis would increase as thoracic kyphosis increases. The thoracic kyphosis angle increases with age and the increase is greater in females than in males [24]. Similar results are recorded in the current study, regarding lumbar lordosis. This increase in lordosis may be attributed to an alteration in the intervertebral discs and a loss in the posterior vertebral body height of lumbar spine. Also, the imbalance in the supporting anterior and posterior soft tissues and musculature might be another contributing factor [25].

Increased lumbar lordosis is one of numerous etiologic factors for low back pain [26]. Also, prolonged sitting is generally accepted as a high risk factor in low back pain; and it is frequently suggested that a lordotic posture of the lumbar spine should be maintained during sitting [27]. Nowadays, measurement of lumbar spine curvature and motion has become common place in the clinical assessment of LBP. It helps in assessment of spinal function and is often used as an outcome measure for clinical intervention studies [28]. The lumbar curvature measurement, as used in Cobb's method [9], may not fully represent the curvature of the spine as shown in some cases of the current study due to differences in posterior inclination of sacrum (Figures 1(b) and 1(c)). Cobb's angle reflects changes in the end vertebrae inclination rather than changes within the spinal curvature; moreover, it neglects the translation of the apical vertebra [29]. Therefore, we added the lordosis index (LI) in assessment. This LI showed significant gender differences in both age groups, with *P* value =0.0066 and <0.0001, for the first and second age groups, respectively. It could be useful in the evaluation of lumbar lordosis, as it depends on the ratio of the breadth (depth) of lumbar curvature and height of the lumbar spine.

Lumbar lordosis is formed by the wedging of the lumbar vertebral bodies and of the intervertebral discs [13]. Lordotic or dorsal wedging (ventral height greater than dorsal height) of the vertebral bodies and the intervertebral discs will increase the LLA, while kyphotic or ventral wedging will decrease it [30]. In the current study, the vertebral bodies as well as the intervertebral discs showed a progressive craniocaudal participation in lumbar lordosis. The vertebral bodies in males showed kyphotic bent in L1, tended to neutral in L2, and then showed progressive lordotic bent from L3 downwards with statistically significant difference between the anterior and posterior heights of the vertebrae. In females, the participation of bodies in lordosis began at higher level, at L2 instead of L3 in males. Similar findings reported that posterior wedging of these vertebrae is about twice as common in females as in males [31]. Bernhardt and Bridwell [32] agreed with the current study that lumbar lordosis usually starts at L1-2 and gradually increases at each level caudally. They added that the lowest three segments account for 80% of the lumbar lordosis. In regard to discs, the current results showed lordotic bent at all levels, progressive in a craniocaudal direction, with maximum lordosis at L5/S1. This trend of increased participation in lumbar lordosis towards caudal segments was also mentioned in other studies [15, 33]. The WI increased by age in the lumbar segments, with statistically significant difference at L5/S1 (*P* = 0.0024).

The lumbar spine is the part of the vertebral column, which is subjected to the compressive load exerted by the incumbent trunk. Its structure is ideally suited to withstand compressive loads [34]. The compressive loads occurred more on the posterior concave aspects, particularly of lower lumbar segments resulting in decrease in the posterior heights and hence increase in lumbar lordosis was noticed in the second age group of the present study (Figures 3(c) and 4(d)).

Despite the X-ray examination being valid and useful for evaluating spinal curvatures, it carries many limitations that include clarifying disc structure and obtaining measurements free from problems due to overlapping of anatomical images [35]. Several studies have proven the accuracy of MRI that has recently become a popular imaging modality, in vertebral measurements, identifying the details of its anatomy [12, 36]. Given its high resolution, it has largely replaced the computed tomography (CT) in the differentiation of the several adjacent structures comprising the spine [36]. We utilized MRI for this study rather than CT scans, because it is more reliable in detecting soft tissue degeneration and hence choosing the cases for study [30]. MRI produces true sagittal tomographic profiles for the spine [37]. In the current study, all cases underwent lumbosacral spine MRI in supine position, with hips and knees flexed, resulting in relative spinal flexion. This position maximizes the dimensions, thus reducing the magnitude of any stenotic effect [38]. Also, it creates a hypolordosis of the lumbar spine relative to the standing position. Positioning the subject in the supine position with extended lower limbs produces the lumbar lordosis of the upright position [39]. In regards to inter- and intraobserver reliability using the interclass correlation coefficient (ICC), the recorded ranges were considered excellent reproducibility. This might render the use of MRI to be more or less an accurate method for study of lumbar spine.

The primary strength of the work was the study of morphology of lumbar lordosis in correlation with other related parameters including the lumbar lordosis angle, lumbar index, and heights of lumbar segments (vertebrae and discs), using highly reliable MRI measures. This is of great value for planning orthopedical surgical procedures, monitoring the progression and treatment of spinal deformities, and determining reference values in normal and pathological conditions [29]. The information is also necessary for constructing accurate mathematical models of the human spine [40]. Such procedures should restore disc height and spine curvature as normally as possible and provide a certain amount of mobility [41].

In conclusion, the study highlights the morphology and dimensions of the lumbar lordosis which represents an important postural factor for sagittal spinal balance. We suggest using WI in association with Cobb's method of LLA in evaluating lumbar curvature. Further studies using MRI are recommended to confirm presence of any association of lordosis with ethnicity and physical activities. Any wide application of the current parameters has to consider the potential limitations of our sampling populations, such as the effect of body height and weight in vertebral angle.

## Figures and Tables

**Table 1:** Definitions of measured lumbar parameters.

	Parameter	Abbreviation	Definition
1	Angle of lumbar lordosis	LLA	The angle between two straight lines passing along the upper border of the body of first lumbar vertebra (L1) and the upper sacral border.
2	Height of lumbar spine curvature	LH	The maximum distance between the upper anterior end of first lumbar vertebra (L1) to that of sacrum.
3	Breadth of lumbar spine curvature	LB	The maximum distance between the deepest point of lumbar curvature (at the back of upper part of L4 body) to the line representing the length of lumbar curvature.
4	Anterior height of lumbar vertebral body	AL (1 to 5)	The maximum distance between superior and inferior limits of the anterior border of lumbar vertebral body at the midsagittal plane.
5	Posterior height of lumbar vertebral body	PL (1 to 5)	The maximum distance between superior and inferior limits of the posterior border of lumbar vertebral body at the midsagittal plane.
6	Anterior height of intervertebral disc	AD (L1/2 to L5/S1)	The maximum distance between superior and inferior limits of the anterior border of lumbar intervertebral disc at the midsagittal plane.
7	Posterior height of intervertebral disc	PD (L1/2 to L5/S1)	The maximum distance between superior and inferior limits of the posterior border of lumbar intervertebral disc at the midsagittal plane.

**Table 2:** Profile of subjects.

	Number	Mean (m)	Standard deviation (SD)
Gender			
Males (M)	46	39.37	±9.09
Females (F)	47	39.60	±9.06
Age groups			
M: 25–41 y	26	32.42	±3.30
42–57 y	20	48.40	±5.44
F: 25–41 y	20	30.35	±4.20
42–57 y	27	46.44	±4.23

**Table 3:** Statistical analysis of the lumbar spine measurements.

	Age	M			F			MD	SE	95% CI	*P* value
		m	SD	R	m	SD	R				
LLA (°)	Total	41.98	6.83	30–60	52.20	4.78	43–67	−10.22	1.22	−12.650 to −7.802	<0.0001
	G1	39.12	5.32	30–50	50.03	4.37	43–60	−10.91	1.45	−13.869 to −7.960	<0.0001
	G2	45.70	6.89	30–60	53.81	4.49	46–67	−8.11	1.66	−11.46 to −4.77	<0.0001

LH (mm)	Total	168.08	6.32	153–184	156.39	7.56	141–170	11.69	1.45	8.815 to 14.562	<0.0001
	G1	170.39	6.24	160–184	159.42	8.25	144–170	10.97	2.14	6.661 to 15.268	<0.0001
	G2	165.09	5.17	153–174	154.15	6.25	141–163	10.94	1.72	7.481 to 14.393	<0.0001

LB (mm)	Total	44.02	4.08	35–51	45.73	4.42	37–58	−1.71	0.88	−3.464 to 0.040	0.0553
	G1	43.23	4.56	35–50.5	43.48	3.63	37–50	−0.24	1.25	−2.753 to 2.265	0.8454
	G2	45.05	3.17	37–51	47.41	4.24	42–58	−2.36	1.13	−4.63 to −0.08	0.0426

LI (%)	Total	26.26	2.46	21–32	29.34	2.97	23–36	−3.08	0.57	−4.21 to −1.95	<0.0001
	G1	25.42	2.32	21–28	27.40	2.35	23–33	−1.98	0.69	−3.37 to −0.58	0.0066
	G2	27.35	2.25	22–32	30.78	2.56	27–36	−3.43	0.72	−4.88 to −1.98	<0.0001

**Table 4:** Statistical analysis of lumbar bodies' anterior (AL) and posterior (PL) heights.

	Age	M			F			MD	SE	95% CI	*P* value
		m	SD	R	m	SD	R				
AL1 (mm)	Total	25.23	1.97	21–30	24.18	1.79	19–26	1.05	0.39	0.275 to 1.824	0.0085
	G1	25.37	2.13	22–30	24.02	1.95	19–26	1.35	0.61	0.119 to 2.582	0.0323
	G2	25.05	1.77	21–28	24.30	1.69	21–26	0.75	0.51	−0.277 to 1.775	0.1486

PL1 (mm)	Total	26.30	26.30	20–30	24.32	24.32	19–28	1.98	0.38	1.221 to 2.745	<0.0001
	G1	26.34	1.55	23–30	24.53	2.36	20–29	1.82	0.58	0.653 to 2.982	0.0030
	G2	26.25	1.90	19–28	24.17	1.70	21–27	2.08	0.53	1.021 to 3.145	0.0003

AL2 (mm)	Total	26.84	1.87	23–32	26.00	2.08	20–30	0.84	0.41	0.021 to 1.653	0.0445
	G1	27.06	1.98	24–32	25.50	2.29	23–29	1.56	0.63	0.288 to 2.828	0.0174
	G2	26.55	1.74	23–29	26.370	1.86	20–30	0.18	0.54	−0.897 to 1.256	0.7384

PL2 (mm)	Total	27.13	1.89	24–30.5	25.09	1.87	20.5–28	2.04	0.39	1.264 to 2.814	<0.0001
	G1	27.25	1.94	24–30	25.37	2.08	20.5–28	1.89	0.60	0.686 to 3.084	0.0028
	G2	26.98	1.87	25–30.5	24.89	1.71	22–28	2.09	0.53	1.029 to 3.143	0.0003

AL3 (mm)	Total	27.29	1.72	24–32	26.98	1.85	22–30.5	0.31	0.37	−0.427 to 1.048	0.4050
	G1	27.67	1.79	25–32	26.59	1.93	22–30.5	1.09	0.55	−0.024 to 2.200	0.0549
	G2	26.80	1.53	24–29	27.28	1.77	24–30.5	−0.48	0.49	−1.472 to 0.516	0.3381

PL3 (mm)	Total	26.90	1.96	23–30	25.15	1.97	21–30	1.75	0.41	0.938 to 2.560	<0.0001
	G1	26.87	2.23	23–30	25.29	1.68	22–28.5	1.58	0.60	0.376 to 2.784	0.0113
	G2	26.95	1.61	22–28.5	25.06	2.19	21–30	1.89	0.58	0.726 to 3.063	0.0021

AL4 (mm)	Total	27.88	1.56	25–31.5	26.91	2.26	23–32	0.97	0.40	0.167 to 1.772	0.0184
	G1	28.23	1.73	26–31.5	26.52	1.84	23–29	1.72	0.53	0.650 to 2.782	0.0023
	G2	27.43	1.22	25–29	27.20	2.52	23–32	0.23	0.61	−1.010 to 1.453	0.7191

PL4 (mm)	Total	25.87	1.84	22.5–30	24.05	2.07	21–30	1.82	0.41	1.011 to 2.622	<0.0001
	G1	26.15	1.95	23–30	24.08	1.48	22–26.5	2.07	0.52	1.024 to 3.133	0.0003
	G2	25.50	1.65	22.5–27.5	24.04	2.44	21–30	1.46	0.63	0.189 to 2.737	0.0254

AL5 (mm)	Total	29.31	1.74	25–33	27.88	2.36	23–32	1.43	0.43	0.567 to 2.280	0.0014
	G1	29.83	1.58	26–33	27.23	2.60	23–31	2.60	0.62	1.358 to 3.854	0.0001
	G2	28.63	1.74	25–31	28.37	2.09	25–32	0.26	0.58	−0.904 to 1.414	0.6603

PL5 (mm)	Total	24.09	1.82	20–27	22.40	2.34	18–28	1.69	0.43	0.818 to 2.544	0.0002
	G1	24.25	1.90	20–27	22.78	1.95	19–25	1.47	0.57	0.318 to 2.624	0.0136
	G2	23.88	1.72	20–27	22.13	2.59	18–28	1.75	0.67	0.401 to 3.089	0.0121

**Table 5:** Statistical analysis of lumbar discs' anterior (AD) and posterior (PD) heights.

	Age	M			F			MD	SE	95% CI	*P* value
		m	SD	R	m	SD	R				
AD1 (mm)	Total	8.91	1.20	7–12.8	8.11	1.17	5.5–10	0.80	0.25	0.316 to 1.293	0.0015
	G1	9.02	1.40	7–12.8	7.89	1.10	5.5–9.5	1.13	0.38	0.372 to 1.904	0.0045
	S2	8.77	0.90	7–10	8.27	1.21	6–10	0.50	0.32	−0.152 to 1.144	0.1301

PD1 (mm)	Total	6.60	1.10	4–9.5	6.69	1.06	5–8.5	−0.09	0.22	−0.532 to 0.358	0.6976
	G1	6.74	1.14	4–9.5	6.64	1.28	5–8.5	0.10	0.36	−0.617 to 0.823	0.7735
	G2	6.42	1.06	5–8	6.72	0.88	5–8	−0.30	0.28	−0.877 to 0.262	0.2829

AD2 (mm)	Total	10.00	1.23	7–12	9.69	1.34	6.7–13	0.31	0.27	−0.221 to 0.838	0.2497
	G1	9.93	1.25	8–12	9.34	1.66	6.7–13	0.59	0.43	−0.271 to 1.455	0.1737
	G2	10.11	1.23	7–12	9.96	0.99	8–12	0.15	0.32	−0.509 to 0.793	0.6626

PD2 (mm)	Total	7.03	0.92	5–9	6.95	1.01	5–9	0.08	0.20	−0.316 to 0.480	0.6850
	G1	7.92	1.47	6–10.5	6.81	1.23	5–9	1.11	0.41	0.289 to 1.937	0.0093
	G2	6.91	0.93	5–9	7.06	0.82	5–8.5	−0.15	0.26	0.667 to 0.366	0.5604

AD3 (mm)	Total	11.30	1.41	8.5–14.5	11.07	1.19	8–13.5	0.23	0.27	−0.302 to 0.770	0.3880
	G1	11.14	1.47	8.5–14	11.08	1.41	8–13	0.06	0.43	−0.811 to 0.921	0.8994
	G2	11.52	1.32	9–14.5	11.06	1.03	9.5–13.5	0.46	0.34	−0.230 to 1.149	0.1861

PD3 (mm)	Total	7.73	1.35	5.5–10.5	7.52	1.23	5.5–9.5	0.21	0.27	−0.326 to 0.736	0.4454
	G1	7.92	1.47	6–10.5	7.53	1.29	5.5–9.5	0.39	0.42	−0.444 to 1.231	0.3493
	G2	7.48	1.14	5.5–10	7.52	1.21	5.5–9	−0.04	0.35	−0.746 to 0.659	0.9013

AD4 (mm)	Total	12.76	1.27	10.5–16	12.51	1.40	8–14.5	0.25	0.28	−0.301 to 0.801	0.3695
	G1	12.83	1.38	10.5–16	12.76	1.41	8–14	0.07	0.41	−0.760 to 0.911	0.8558
	G2	12.68	1.14	11–15	12.33	1.39	10–14.5	0.35	0.38	−0.426 to 1.109	0.3749

PD4 (mm)	Total	7.87	1.22	6–11	8.01	1.27	5.5–10	−0.15	0.26	−0.659 to 0.368	0.5753
	G1	8.20	1.17	6.5–11	8.28	1.34	5.5–10	−0.08	0.37	−0.825 to 0.665	0.8297
	G2	7.44	1.18	6–9	7.82	1.21	5.5–9	−0.38	0.35	−1.090 to 0.331	0.2874

AD5 (mm)	Total	14.41	1.55	11–18	13.97	1.80	10.5–17	0.44	0.35	−0.249 to 1.138	0.2057
	G1	14.25	1.44	11–16.5	14.55	1.50	10.5–16	−0.30	0.44	−1.179 to 0.579	0.4951
	G2	14.63	1.71	11–18	13.54	1.91	10.5–17	1.09	0.54	0.003 to 2.172	0.0493

PD5 (mm)	Total	6.82	0.96	5–9	6.73	1.16	4.5–9	0.09	0.22	−0.357 to 0.520	0.7140
	G1	7.37	0.82	6–9	7.03	0.99	4.5–8.5	0.34	0.27	−0.198 to 0.879	0.2094
	G2	6.10	0.60	5–7	6.52	1.24	5–9	−0.42	0.30	−1.023 to 0.186	0.1699

**Table 6:** Statistical analysis of wedge indices (WI) of lumbar spine segments; lumbar bodies (L); and intervertebral discs (L/) in gender groups.

	Age	M			F			MD	SE	95% CI	*P* value
		m	SD	R	m	SD	R				
L1	Total	95.93	4.69	85–108	99.66	5.57	91–119	−3.72	1.07	−5.85 to −1.60	0.0008
	G1	96.19	4.54	88–107	98.30	3.61	93–106	−2.11	1.24	−4.61 to 0.39	0.0961
	G2	95.60	4.97	85–108	100.67	6.55	91–119	−5.07	1.75	−8.59 to −1.54	0.0058

L2	Total	98.98	3.96	92–112	103.77	7.11	92–123	−4.79	1.20	−7.16 to −2.41	0.0001
	G1	99.35	3.39	92–100	100.65	4.32	92–107	−1.30	1.14	−3.59 to 0.99	0.2575
	G2	98.50	4.65	92–112	106.07	7.92	94–123	−7.57	1.99	−11.58 to −3.57	0.0004

L3	Total	101.78	4.24	78–109	107.57	5.30	99–118	−5.79	1.00	−7.77 to −3.81	<0.0001
	G1	103.23	3.99	78–109	105.35	4.51	99–113	−2.12	1.08	−4.27 to 0.03	0.0536
	G2	99.90	3.86	88–105	109.22	5.31	100–118	9.32	1.40	−12.15 to −6.50	<0.0001

L4	Total	108.04	5.76	100–120	111.74	8.03	98–130	−3.70	1.45	−6.59 to −0.82	0.0125
	G1	108.23	6.33	100–117	109.45	8.74	98–122	−1.22	2.22	−5.69 to 3.26	0.5857
	G2	107.80	5.07	100–120	113.44	7.16	103–130	−5.64	1.88	−9.43 to −1.86	0.0043

L5	Total	122.17	9.87	108–165	125.30	12.56	96–152	−3.12	2.35	−7.78 to 1.54	0.1862
	G1	123.65	10.47	108–165	120.00	11.85	96–135	3.65	3.30	−2.99 to 10.30	0.2738
	G2	120.25	8.92	108–140	129.22	11.78	108–152	−8.97	3.15	−15.31 to −2.63	0.0066

L1/2	Total	137.02	20.97	100–200	124.68	26.02	86–182	12.34	4.91	2.59 to 22.09	0.0136
	G1	135.73	20.40	100–200	123.20	23.98	88–160	12.53	6.55	−0.67 to 25.73	0.0622
	G2	138.70	22.11	100–180	125.78	27.83	86–182	12.92	7.54	−2.27 to 28.11	0.0936

L2/3	Total	143.41	21.05	113–200	141.36	20.58	100–190	2.05	4.32	−6.52 to 10.63	0.6358
	G1	139.77	18.75	113–176	138.90	20.79	106–190	0.87	5.85	10.91 to 12.65	0.8825
	G2	148.15	23.34	121–200	143.19	20.63	100–183	4.96	6.44	−8.00 to 17.93	0.4445

L3/4	Total	148.89	22.42	110–207	150.38	26.45	118–237	−1.49	5.09	−11.60 to 8.62	0.7701
	G1	142.54	15.84	110–169	148.95	18.72	127–183	−6.41	5.10	−16.69 to 3.86	0.2151
	G2	157.15	27.08	113–207	151.44	31.28	118–237	5.71	8.73	−11.87 to 23.28	0.5166

L4/5	Total	165.13	24.84	110–217	159.87	30.72	122–236	5.26	5.80	−6.26 to 16.78	0.3670
	G1	158.69	24.09	110–215	156.85	25.60	123–236	1.84	7.36	−12.99 to 16.68	0.8036
	G2	173.50	23.81	141–217	162.11	34.33	122–236	11.39	8.95	−6.64 to 29.41	0.2097

L5/S1	Total	214.85	38.21	133–320	212.43	39.70	131–340	2.42	8.08	−13.63 to 18.48	0.7651
	G1	195.08	22.88	133–233	209.80	26.32	153–260	−14.72	7.26	−29.36 to −0.08	0.0487
	G2	240.55	39.24	187–320	214.37	47.68	131–340	26.18	13.07	−0.15 to 52.51	0.0513

**Table 7:** Statistical analysis of wedge indices (WI) of lumbar spine segments; lumbar bodies (L); and intervertebral discs (L/) in both age groups.

	Group 1 (number 46)			Group 2 (number 47)			MD	SE	95% CI	*P* value
	m	SD	R	m	SD	R				
L1	97.11	4.25	88–107	98.51	6.39	85–119	−1.40	1.13	−3.64 to 0.84	0.2173
L2	99.91	3.83	92–110	102.85	7.66	92–123	−2.94	1.26	−5.44 to −0.43	0.0220
L3	104.15	4.31	99–113	105.26	6.62	88–118	−1.10	1.16	−3.41 to 1.20	0.3445
L4	108.76	7.41	98–122	111.04	6.90	100–130	−2.28	1.48	−5.23 to 0.67	0.1276
L5	122.07	11.11	98–122	125.40	11.47	96–165	−3.34	2.34	−7.99 to 1.31	0.1574
L1/2	130.28	22.66	88–200	131.28	26.10	86–182	−0.99	5.07	−11.07 to 9.08	0.8451
L2/3	139.39	19.44	106–190	145.30	21.72	100–200	−5.91	4.28	−14.40 to 2.59	0.1707
L3/4	145.33	17.25	110–183	153.87	29.40	113–237	−8.55	5.01	−18.50 to 1.41	0.0916
L4/5	157.89	24.49	110–236	166.96	30.54	122–236	−9.07	5.75	−20.48 to 2.35	0.1182
L5/S1	201.48	25.25	133–260	225.51	45.74	131–340	−24.03	7.69	−39.30 to −8.77	0.0024

## References

[1] Jang J-S, Lee S-H, Min J-H, Maeng DH (2009). Influence of lumbar lordosis restoration on thoracic curve and sagittal position in lumbar degenerative kyphosis patients. *Spine*.

[2] Been E, Kalichman L (2014). Lumbar lordosis. *The Spine Journal*.

[3] Chang K, Leng X, Zhao W (2011). Quality control of reconstructed sagittal balance for sagittal imbalance. *Spine*.

[4] Lin R, Lee R, Huang Y, Chen S, Yu C (2002). Analysis of lumbosacral lordosis using standing lateral radiographs through curve reconstruction. *Biomedical Engineering—Applications, Basis and Communications*.

[5] Hong CH, Park JS, Jung KN, Kim WJ (2010). Measurement of the normal lumbar intervertebral disc space using magnetic resonance imaging. *Asian Spine Journal*.

[6] Parkin IG, Harrison GR (1985). The topographical anatomy of the lumbar epidural space. *Journal of Anatomy*.

[7] Cilliers A, Schulenburg DH, van Rensburg JJ, Gen D (2010). MRI determination of the vertebral termination of the dural sac tip in a South African population: clinical significance during spinal irradiation and caudal anaesthesia. *SA Journal of Radiology*.

[8] Goh S, Tan C, Price RI (2000). Influence of age and gender on thoracic vertebral body shape and disc degeneration: an MR investigation of 169 cases. *Journal of Anatomy*.

[9] Harrison DE, Cailliet R, Harrison DD, Janik TJ, Holland B (2001). Reliability of centroid, Cobb, and Harrison posterior tangent methods: which to choose for analysis of thoracic kyphosis.. *Spine*.

[10] Cronbach LT, Gleser GC, Nanda H, Rajaratnam N (1972). *The Dependability of Behavioral Measurements: Theory of Generalizability for Scores and Profiles*.

[11] Voutsinas SA, MacEwen GD (1986). Sagittal profiles of the spine. *Clinical Orthopaedics and Related Research*.

[12] Matsumoto M, Okada E, Kaneko Y (2011). Wedging of vertebral bodies at the thoracolumbar junction in asymptomatic healthy subjects on magnetic resonance imaging. *Surgical and Radiologic Anatomy*.

[13] Vialle R, Levassor N, Rillardon L, Templier A, Skalli W, Guigui P (2005). Radiographic analysis of the sagittal alignment and balance of the spine in asymptomatic subjects. *Journal of Bone and Joint Surgery A*.

[14] Jackson RP, McManus AC (1994). Radiographic analysis of sagittal plane alignment and balance in standing volunteers and patients with low back pain matched for age, sex, and size: a prospective controlled clinical study. *Spine*.

[15] Damasceno LHF, Catarin SRG, Campos AD, Defino HLA (2006). Lumbar lordosis: a study of angle values and of vertebral bodies and intervertebral discs role. *Acta Ortopédica Brasileira*.

[16] Murrie VL, Dixon AK, Hollingworth W, Wilson H, Doyle TAC (2003). Lumbar lordosis: study of patients with and without low back pain. *Clinical Anatomy*.

[17] Stagnara P, de Mauroy JC, Dran G (1982). Reciprocal angulation of vertebral bodies in a sagittal plane: approach to references for the evaluation of kyphosis and lordosis. *Spine*.

[18] Nourbakhsh MR, Moussavi SJ, Salavati M (2001). Effects of lifestyle and work-related physical activity on the degree of lumbar lordosis and chronic low back pain in a Middle East population. *Journal of Spinal Disorders*.

[19] Snell R (2012). *Clinical Anatomy by Regions*.

[20] Tüzün C, Yorulmaz I, Cindaş A, Vatan S (1999). Low back pain and posture. *Clinical Rheumatology*.

[21] Lee ES, Ko CW, Suh SW, Kumar S, Kang K, Yang JH (2014). The effect of age on sagittal plane profile of the lumbar spine according to standing, supine, and various sitting positions. *Journal of Orthopaedic Surgery and Research*.

[22] Cheng XG, Sun Y, Boonen S (1998). Measurements of vertebral shape by radiographic morphometry: Sex differences and relationships with vertebral level and lumbar lordosis. *Skeletal Radiology*.

[23] Ghandhari H, Hesarikia H, Ameri E, Noori A (2013). Assessment of normal sagittal alignment of the spine and pelvis in children and adolescents. *BioMed Research International*.

[24] Nishiwaki Y, Kikuchi Y, Araya K (2007). Association of thoracic kyphosis with subjective poor health, functional activity and blood pressure in the community-dwelling elderly. *Environmental Health and Preventive Medicine*.

[25] De Smet AA, Robinson RG, Johnson BE, Lukert BP (1988). Spinal compression fractures in osteoporotic women: patterns and relationship to hyperkyphosis. *Radiology*.

[26] Kim H, Chung S, Kim S (2006). Influences of trunk muscles on lumbar lordosis and sacral angle. *European Spine Journal*.

[27] Lengsfeld M, Frank A, van Deursen DL, Griss P (2000). Lumbar spine curvature during office chair sitting. *Medical Engineering and Physics*.

[28] Williams JM, Haq I, Lee RY (2010). Dynamic measurement of lumbar curvature using fibre-optic sensors. *Medical Engineering and Physics*.

[29] Vrtovec T, Pernuš F, Likar B (2009). A review of methods for quantitative evaluation of spinal curvature. *European Spine Journal*.

[30] Lakshmanan P, Purushothaman B, Dvorak V, Schratt W, Thambiraj S, Boszczyk BM (2012). Sagittal endplate morphology of the lower lumbar spine. *European Spine Journal*.

[31] Ericksen MF (1978). Aging in the lumbar spine. II. L1 and L2. *American Journal of Physical Anthropology*.

[32] Bernhardt M, Bridwell KH (1989). Segmental analysis of the sagittal plane alignment of the normal thoracic and lumbar spines and thoracolumbar junction. *Spine*.

[33] Gelb DE, Lenke LG, Bridwell KH, Blanke K, McEnery KW (1995). An analysis of sagittal spinal alignment in 100 asymptomatic middle and older aged volunteers. *Spine*.

[34] Rajnics P, Pomero V, Templier A, Lavaste F, Illes T (2001). Computer-assisted assessment of spinal sagittal plane radiographs. *Journal of Spinal Disorders*.

[35] Tarantino U, Fanucci E, Iundusi R (2013). Lumbar spine MRI in upright position for diagnosing acute and chronic low back pain: statistical analysis of morphological changes. *Journal of Orthopaedics and Traumatology*.

[36] Jindal G, Pukenas B (2011). Normal spinal anatomy on magnetic resonance imaging. *Magnetic Resonance Imaging Clinics of North America*.

[37] Roberts N, Gratin C, Whitehouse GH (1997). MRI analysis of lumbar intervertebral disc height in young and older populations. *Journal of Magnetic Resonance Imaging*.

[38] Alyas F, Connell D, Saifuddin A (2008). Upright positional MRI of the lumbar spine. *Clinical Radiology*.

[39] Andreasen ML, Langhoff L, Jensen TS, Albert HB (2007). Reproduction of the lumbar lordosis: A comparison of standing radiographs versus supine magnetic resonance imaging obtained with straightened lower extremities. *Journal of Manipulative and Physiological Therapeutics*.

[40] Panjabi MM, Goel V, Oxland T (1976). Human lumbar vertebrae. Quantitative three-dimensional anatomy. *Spine*.

[41] Schwab F, Lafage V, Boyce R, Skalli W, Farcy J (2006). Gravity line analysis in adult volunteers: age-related correlation with spinal parameters, pelvic parameters, and foot position. *Spine*.

